# Exploring how national educational organizations can promote educational research amongst members: a survey-based study

**DOI:** 10.1186/s12909-022-03202-3

**Published:** 2022-03-02

**Authors:** Lavjay Butani, Gary L. Beck Dallaghan

**Affiliations:** 1 Department of Pediatrics, University of California Davis School of Medicine, 2516 Stockton Blvd, 95817 Sacramento, CA USA; 2 Office of Medical Education, University of North Carolina School of Medicine, 108 Taylor Hall, 27599 Chapel Hill, NC USA

**Keywords:** Educational research, Survey, Undergraduate medical education, Organizational strategies, Faculty

## Abstract

**Background:**

Engagement of academic faculty in research remains low. While barriers to research have been explored, there are no data on how national organizations can help overcome these barriers. Our study explored faculty satisfaction and motivational drivers for engagement with research opportunities offered by the Council on Medical Student Education in Pediatrics (COMSEP), an organization of pediatric medical educators, and characterize strategies perceived by faculty to promote the use of these opportunities.

**Methods:**

In 2021, 5 survey questions were administered to faculty members of COMSEP to explore satisfaction with COMSEP’s research offerings, the perceived value of educational research, and the facilitators, barriers and potential opportunities for COMSEP to promote research. Clark’s Commitment and Necessary Effort model on motivation served as the theoretical framework for our study, which explores motivation, self-efficacy and contextual factors influencing an individual’s pursuit of goals. Chi-square analysis and Wilcoxon Signed Ranks Test were used to compare categorical and scaled variables among groups who did and did not avail of COMSEP’s research offerings.

**Results:**

90 (25%) of 360 recipients responded. 61% expressed satisfaction with COMSEP’s research offerings. 68% indicated research was an expectation of their academic appointment, that education was their primary research focus (74%) and that they did not have other research opportunities that met their needs (58%). Of respondents, 75.7% of females had submitted a proposal compared to 60% of non-responders who were females. The comparison by gender was not statistically significant. Exploration by academic rank revealed that 35% of instructor/assistant professors had submitted a proposal compared to 65% of associate professors/professors (*p* =.05). Barriers leading to non-submission to any of the offerings included having too much other work, lack of enjoyment in writing and inability to find mentors. Respondents endorsed the importance of several strategies to promote engagement in research-skill building opportunities, personalized consultations and increased funding.

**Conclusions:**

Faculty educators *value* the importance of educational research and recognize that research opportunities offered by COMSEP address an unmet need, but express ambivalence in the enjoyment of writing (reflecting their *mood*), and endorse structural barriers, that are amenable to change, affecting their *personal agency*.

**Supplementary Information:**

The online version contains supplementary material available at 10.1186/s12909-022-03202-3.

## Background

Engagement of academic faculty in research activities remains low. Studies on research productivity and engagement among pediatric residents [1], fellows in training [2] and faculty [3, 4] have all identified many challenges that researchers face. Only a small fraction of oral and poster presentations by faculty at national educational meetings make it to print [5]. While there are some data exploring barriers faced by faculty in converting such presentations at national meetings into publications [6], there are no data on whether and how national educational organizations can help overcome barriers that members may face in taking advantage of research opportunities that are offered, in the first place.

There is literature to suggest that institutional factors may play a larger role than individual factors in promoting scholarship [7]. A consistent set of 12 characteristics was found in research-conducive environments: clear goals that serve a coordinating function, research emphasis, distinctive culture, positive group climate, assertive participative governance, decentralized organization, frequent communication, accessible resources, particularly human, sufficient size, age, and diversity of the research group, appropriate rewards, concentration on recruitment and selection, and leadership with research expertise and skill in both initiating appropriate organizational structure and using participatory management practices [8]. Based on these principles, a few educational research groups have been created, with the primary goal of promoting educational scholarship [9, 10], with preliminary outcome data suggesting some gains in research output.

The Council on Medical Student Education in Pediatrics (COMSEP) a well-established organization of pediatric undergraduate medical educators in North America, has been a leader in pediatric undergraduate medical education (UME) since the early 1990’s [11] and one that has *“advancing the art and science of medical student education in pediatrics”* as one of its main goals (https://www.comsep.org/about-comsep/). To promote educational research, one of COMSEP’s many educational missions, in addition to the Research and Scholarship Collaborative (a group that is to open to all members and one that attempts to facilitate medical educational scholarship through periodic meetings), the organization offers 3 programs that are competitive and have the greatest potential to lead to a tangible product or be recognized as a form of educational research. These are the Grant Program that offers funding for a limited number of educational research projects by members on an annual basis, the Annual Survey that enables applicants to ask members questions that have the potential to lead to a research manuscript, and lastly the COMSEP feature article published in *Pediatrics* that addresses core clinical skills that educators and busy clinicians may benefit from in their teaching efforts.

The aim of our study was to explore the satisfaction of faculty with the aforementioned research offerings of COMSEP and to explore facilitators and barriers faced by members in availing of these options. We hypothesized that there are likely individual factors as well as organizational strategies that can be pursued, and resources be provided to members, that may help promote educational research, while at the same time continuing to support the other missions of COMSEP. Understanding these may help increase member satisfaction and research productivity, both of which could promote academic advancement.

As a theoretical framework, we used Clark’s Commitment and Necessary Effort (CANE) model of motivation [12] to explore various facets of faculty commitment and pursuit of educational research and satisfaction with the research offerings by COMSEP. The model defines motivation (the process whereby goal-directed activity is instigated and sustained) as having two components: commitment and necessary effort. Commitment refers to actively pursuing a goal over time in the face of distractions and effort is the amount and quality of energy people invest in achieving the knowledge component of performance goals. The 3 factors influencing motivation are ***personal agency ****(“can I do this?”*) which is a product of self-efficacy and contextual factors, ***mood or emotion**** (“do I feel like doing this?*”) and ***value*** (“will this be of help?”, “is this who I am?” or “am I curious about it?”). Evaluating issues related to task completion using this framework can help address both motivational barriers and organizational factors that may help guide potential solutions.

## Methods

This was a survey-based study using data from the 2021 annual member survey of COMSEP. The COMSEP Annual Survey is a platform to investigate topics of relevance to its members and includes multiple topical surveys limited to a specified number of questions approved by the COMSEP Annual Survey Committee. The overall study population for the survey included all members of COMSEP, comprised of leaders in pediatric UME in the US and Canada, such as clerkship directors, clinical site directors, teaching faculty and administrators; for the purpose of our research question, only non-emeritus faculty members of COMSEP constituted the study population. Emeritus faculty membership is open only to faculty who have retired; since a major goal of our study was to explore factors that motivate faculty to engage in research and investigate facilitators and barriers to research, we chose to include only faculty who are actively engaged in work at their respective institutions. The anonymously administered web-based survey was distributed by the COMSEP Annual Survey Committee; after the initial invitation to complete the survey, reminder emails were sent until the survey was closed three months later.

The 2021 COMSEP Annual Survey gathered information about respondents’ institutional affiliations, as well as other relevant topics in pediatric UME. In addition, 5 questions related to educational research were included. After each question, an open-ended comment box was provided for respondents to elaborate on their answers, if they so desired. Questions were developed by the investigators and were based on a comprehensive literature review related to educational research and its facilitators and barriers. The survey questions were pilot tested at the investigators’ respective institutions; additional feedback was provided by the members of the Annual Survey Committee and the Executive Committee of COMSEP, all with expertise in pediatric medical education. In all, three iterations of pilot testing were performed; with each iteration, pilot testers were asked to confirm that the questions were easy to understand and complete. Pilot testers were queried about whether there was a need for definitions to understand the questions, the clarity of instructions and the lack of ambiguity in the question stems and answer choices. Based on feedback some questions were eliminated and others were consolidated, and the phrasing of a few questions was refined. The final set of questions was then incorporated into the web survey. Survey completion was voluntary and there were no incentives to participate. Data was collected by COMSEP and made available to investigators for analysis.

Survey questions, with links to the theoretical framework used, and response rates, are available as supplemental online material.

Quantitative data were analyzed using descriptive statistics. Chi-square analysis and Wilcoxon Signed Ranks Test were used to analyze categorical and scaled variables among groups. Statistical analyses were conducted using IBM SPSS v 28 (Armonk, NY). Due to the small number of comments entered into the comment boxes, the investigators chose not to analyze the data using a qualitative content analysis approach; rather, comments representative of the quantitative data were chosen and are presented verbatim.

 The study was granted an exempt status by the Institutional Review Board of the University of California Davis. A waiver for obtaining written informed consent from study participants was granted; completion of survey questions was presumed to express consent by participants.

## Results

Of the 360 non-emeritus faculty who received the survey, 90 (25%), representing at least 28% of all DO and MD-degree granting accredited medical schools in the US and Canada, answered our questions. 67% (61/90) of the respondents were women; 4% (4/90) were instructors, 43% (39/90) were assistant professors, 24.4% (22/90) were associate professors and 27.8% (25/90) were professors. The majority of respondents had defined administrative roles in undergraduate medical education- by far the largest group was comprised of clerkship directors (CD) and associate CDs (48/90; 53%).

### Satisfaction with research offerings of COMSEP

Sixty one percent (55/90) of respondents agreed or strongly agreed (6.7% disagreed) that they were satisfied with the opportunities for educational research that COMSEP offers. A representative comment was *“There is a lot of collaboration across institutions that occurs within COMSEP, more than other organizations I have been a part of. That allows to overcome some of the difficulties of medical education research in not having enough participants in a study.”* While several respondents commented that they were satisfied with the variety of offerings and had benefited from them, some others expressed a lack of awareness of the research opportunities that were available to them and suggested that the organization needed to do a better job in disseminating information about these offerings: *“These opportunities should really be highlighted and pushed - it is the most helpful way the organization can help with academic promotion of its members. I have no clue what opportunities exist and I would say I read most COMSEP emails/blasts/etc.”*

### Interest and research expectations

Recognizing that faculty have diverse interests and responsibilities and that different faculty academic tracks have different expectations for research, respondents were asked to rate their level of agreement with a series of statements exploring this. As can be seen from Table 1, most respondents endorsed research as being an expectation as part of their academic appointment (67.8%) and that medical education was their primary research focus (74.4%). Most respondents (57.8%) disagreed with the statement that they had other research opportunities outside of what COMSEP offered (*“I have dual interests-med ed and clinical and do research on both, I do have some other AAMC offerings that I have used, but COMSEP complements these”)* but also endorsed having too many other responsibilities (50%) to focus on research. One respondent commented: “M*y institution requires this for promotion. I don’t have the necessary protected time - or I am really bad at knowing how to protect my time - to accomplish this. Probably a little bit of both.”*

**Table 1:** Research expectations for academic track

Statement	Level of agreement (agree/strongly agree)	Level of disagreement (disagree/strongly disagree)
Research is not expected for my academic track	21% (19/90)	67.8% (61/90)
Research is expected for my academic track, but I have too many other responsibilities to focus on this aspect of my work.	50% (45/90)	32% (29/90)
Research is expected for my academic track and my primary research focus is not in medical education	8.9% (8/90)	74.4% (67/90)
Research is expected for my academic track and my primary research focus is in medical education, but I have other research opportunities, offered outside of COMSEP, that meet my needs.	20% (18/90)	57.8% (52/90)

### Faculty experience in availing of research opportunities offered by COMSEP

Respondents were then primed to think about the following 3 research offerings of COMSEP (Grants Program, *Pediatrics* feature, and the Annual Survey). These 3 opportunities were chosen since they are long-standing competitive offerings, recur on a regular basis (at least annually), and frequently lead to, or have the greatest potential to lead to, a tangible scholarly product that is a traditional requirement to meet the criteria for ‘research’ by faculty promotions committees [13].

**Fig. 1:**
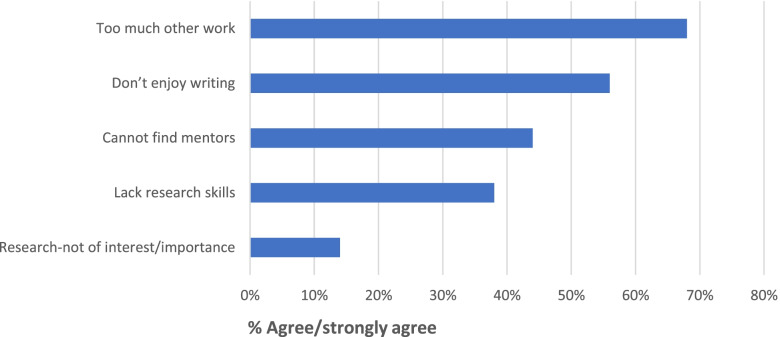
Reasons for non-submissions to research offerings

Fifty five percent (50/90) of respondents stated that they had never submitted a proposal or pre-proposal to any of these 3 offerings. To better understand why respondents had never submitted a proposal or pre-proposal, they were asked to rate their level of agreement with a series of statements. As can be seen in Fig. 1 and in alignment with the prior response, the most common reason for non-submission was lack of time due to other work/responsibilities (68%). Other factors contributing to non-submission by a substantial number of respondents included lack of enjoyment in writing manuscripts or grants (56%), inability to find mentors to take research ideas to the next step (44%) and lack of research skills (38%). No comments in the open-ended comment box highlighted any other areas of concern by respondents.

### Feedback on the implementation of COMSEP’s research offerings

A series of questions was posed to seek feedback from the 40 faculty who had taken advantage of one or more of COMSEP’s research offerings. 92% (37/40) agreed or strongly agreed that they were satisfied with the submission process and outcome and none expressed disagreement with that sentiment. Similarly, 90% (36/40) expressed agreement or strong agreement with their satisfaction with the review process (1/40; 2.5% disagreed) with the majority, although fewer (29/40; 72.5%), also expressing agreement/strong agreement with the feedback they received; 4/40 (10%) disagreed with this.

### Organizational opportunities to increase educational research

Respondents were posed a final set of questions to understand if COMSEP could do something more or differently that would facilitate their participation in educational research, and were asked to rate the importance of a series of actions that could be considered by the organization. Across the board, respondents reported that all 5 suggestions (Table 2) put forward were of importance to them in increasing their involvement in educational research; the two potential suggestions that appealed to the largest number of respondents were making consultants available to help with their research design/ideas (94% rated that as being important/extremely important) and offering more skill building opportunities for manuscript writing and research (91% rated that as being important/extremely important).

**Table 2:** Organizational opportunities to promote research

Statement	Level of importance (important/extremely important)
Offer more research or manuscript writing skill-building opportunities	91% (82/90)
Offer personalized consultation on research ideas, submissions and pre-submissions	89% (80/90)
Make available consultants with research expertise to help with study design	94% (85/90)
Increase funding opportunities for research	70% (63/90)
Improve how the current offerings are structured/implemented	67% (60/90)

Comments from respondents echoed the quantitative data and also emphasized the benefits of working as part of collaborative research endeavors. The experiences with collaboration in COMSEP have been highly variable as can be seen in these quotes:

*“Having a personalized mentor that can help bring your ideas of research into fruition would be amazing. It would also be good to have people who have similar interests to work together to do multi-institutional research projects. I know there is a research collaborative, so perhaps one of the responsibilities of that collaborative is to help other collaboratives (or individuals), by mentoring them through the research process. I’m sure that is already done to some extent, but perhaps could be more explicit?*”

*“…the projects I have been involved on within my home institution have been as a part of a team. I have never led a project and not confident in my skills to do so. I think the mentoring in the course of a real study is the best way to gain the skills. This may be happening with members of the research collaborative. I am involved with another collaborative and haven’t really thought about how I can utilize the research collaborative to support my work, as I don’t feel I have the expertise to contribute to the work of the collaborative.”*

### Comparisons of those who have submitted proposals to non-submitters

Comparisons were made between those who had submitted proposals versus those who had not. Of respondents, 75.7% of females had submitted a proposal (compared to the non-submitter group that had 60% as females). The comparison by gender was not statistically significant (X^2^_1_=2.35, *p* >.05). Exploration by academic rank revealed that 35% of instructor/assistant professors had submitted a proposal compared to 65% of associate professors/professors. The comparison by academic rank bordered on statistical significance (X^2^_2_=5.880, *p* =.05). Scaled questions were analyzed using Wilcoxon Signed Rank Tests to compare responses between those who submitted proposals compared to non-submitters. There was a statistically significant difference between the groups for those whose primary focus was non-medical education research (z= -3.095, *p* =.002). All other comparisons were not statistically significant (Table 3).

**Table 3:** Comparing data between those who had submitted a proposal and non-submitters

	Submitters	Non-submitters	z-value	*p* value*
	**% respondents who agreed/strongly agreed**			
Research is expected for track	17.5%	24%	-1.586	0.113
Research expected but too much other work	47.5%	52%	-0.742	0.458
Non-education research focus	5%	12%	-3.095	0.002
Non-COMSEP research opportunities available	17.5%	22%	-0.252	0.801
More skill building needed	92.5%	90%	0.000	1.00
Personalized consultation	92.5%	86%	-1.755	0.079
Need for study design experts	95%	94%	-0.734	0.463
Increased funding	80%	62%	-1.220	0.222
Improve current offerings	60%	72%	-0.947	0.344

* Groups were compared using Wilcoxon Signed Rank Tests; data are presented as % for ease of interpretation

## Discussion

Using the CANE model of motivation to interpret our findings, our study explored potential sources of barriers that UME faculty educators face or perceive exist, that could impede their engagement in educational research. Our key findings indicate that although faculty members of COMSEP expressed satisfaction with the research offerings available to them and their implementation, they identified several areas of improvement that can serve as opportunities for the organization to better meet member needs. First and foremost was the observation by a few respondents indicating the need to more prominently highlight these research opportunities and remind members, especially new members, of their existence and of their potential. That the organization should continue to offer these and potentially other research opportunities was reinforced by the responses of the majority of respondents, indicating that research was an expectation for their academic track, that education was their primary research focus and that they did not have other educational research opportunities that met their needs. As can be expected, those with a non-education research focus were less likely to have availed of any of the research offerings of COMSEP since they presumably had other areas of research interest and research needs that COMSEP would not be well suited to meet. Another observation pertained to the need for greater coordination and collaboration among the organization’s various collaboratives so as to provide a fertile ground where research ideas could be generated and steps taken to bring these ideas to fruition using the shared expertise of members.

Through the lens of the CANE model, our study suggests that faculty educators *value* educational research and are interested in it (only 14% of non-submitters disagreed with this statement). However, there appear to be personal and organizational level barriers that impede the engagement of faculty in educational research. At a personal level, many faculty (56%) who had not availed of the research opportunities of COMSEP expressed a lack of enjoyment derived from writing manuscripts or grants (affecting their *mood/emotion*). Whether this is a consequence of their limited experience/expertise or lack of mentorship and amenable to change is unclear and worth exploring in future studies. Many barriers were highlighted that affected faculty *personal agency* such as too much administrative work (affecting self-efficacy) and lack of research skills along with an inability to find mentors, both of which pose contextual barriers. The observation that more senior faculty members were more likely to have availed of the research opportunities offered by COMSEP may be explained by the acquisition of research skills by senior faculty learned ‘on the job’ increasing their personal agency; this observation also highlights the importance of using experienced faculty as valuable mentors for more junior faculty.

Many of the limitations of our study are common in survey-based studies such as a low response rate and social desirability bias affecting responses. The observation that respondents in our study were almost equally divided into groups that had and had not ever availed of the research offerings of COMSEP, provides some indication that there was a measure of balance and diversity in sampling among those who completed the survey leading to a good level of understanding of faculty needs with respect to research. In addition, we chose to focus solely on educational research and not on other forms of educational scholarship, which are increasingly being acknowledged by academic institutions as worthy of recognition in the process of faculty advancements. Our study and findings in no way indicate or reflect our opinion that that all faculty must engage in educational research, rather, our goal is to identify ways to promote research among those who want to and/or need to. Nevertheless, our results should be interpreted with caution, due to the low response rate.

## Conclusions

Our findings indicate the need for COMSEP and similar education-focused organizations to take steps to mitigate barriers that prevent such faculty from engaging in research since this can impede their professional development, satisfaction and advancement, many of which are aligned with the 12 characteristics of effective research organizations put forth by Bland et al. [8]. Similar barriers have been identified at the level of pediatric learners at different levels (residents and fellows in training) and by faculty including lack of time, inadequate funding and paucity of skilled mentors [1, 2, 4]. Respondents identified several strategies to help address these barriers. These and others, based on our findings could include the following:

1. Writing groups to help promote skill building, mentorship and accountability in writing, leading to the generation of a positive *mood/emotion* towards grant submissions and manuscript writing [14–16]. These could be built into the existing collaboratives within the organization or be a distinct cross-collaborative group. Having a mix of faculty of different ranks and experiences/expertise could further help promote research since in our study, those at a higher rank were more likely to have submitted a proposal/pre-proposal.
2. Increasing other skill building opportunities that can also build *self-efficacy* such as workshops, training programs (at various learner levels including during medical school, residency and fellowship training), and pairing more experienced faculty with those less familiar with research methodology.
3. Organizational strategies to provide additional support to faculty such as increased collaboration among the various collaboratives, increased funding opportunities, personalized consultations with those skilled in research methodology and grant writing (including having a focused funding mechanism for clearly defined consultation needs) and providing research mentorship across all collaborative groups, all of which can also help build *self-efficacy*, and lastly.
4. Advocacy at the organizational level by collaborating with other like-minded groups, such as residency program directors, Department chairs and Medical School Deans to provide ample time for faculty to engage in educational research and finding strategies to move some of the administrative load involved in UME to others. While this has been an ongoing effort and one that the Alliance for Clinical Education has promoted, much still needs to be done to ensure that sufficient time be provided to educational leaders to allow them to effectively engage in educational research [17].

 

## Supplementary Information

**Additional file 1.** Survey Questions with Links to Theoretical Framework

## Data Availability

The datasets used and/or analyzed during the current study are available from the corresponding author on reasonable request.

## Abbreviations

CANE: Commitment and Necessary Effort
CD: Clerkship director
COMSEP: Council on Medical Student Education in Pediatrics
UME: Undergraduate medical education
