# Supplementary material for: Exploring how national educational organizations can promote educational research amongst members: a survey-based study
Source: BMC Med Educ. 2022 Mar 2;22:137. doi: 10.1186/s12909-022-03202-3 (PMC8889650; doi:10.1186/s12909-022-03202-3)
Supplement: Supplementary file 1 — Additional file 1. Survey Questions with Links to Theoretical Framework [file 12909_2022_3202_MOESM1_ESM.docx]

| **Objective and link to theoretical framework** | **Question** | **Response format** | **Number of respondents** |
| --- | --- | --- | --- |
| 1) Satisfaction with current research offerings  (affects *mood* and may reflect *contextual factors*) | COMSEP offers several educational research opportunities for members that can lead to a peer-reviewed manuscript.  Please rate your level of agreement with the following statement ‘I am satisfied with the educational research opportunities that COMSEP offers’ | Strongly agree  Agree  Neither agree nor disagree  Disagree  Strongly disagree | 90 |
| 2) Importance given to educational research  (explores *value* given to educational research offerings) | Faculty have diverse interests and responsibilities; moreover, different faculty academic tracks have different expectations for research.  Please rate your level of agreement with the following set of statements.  1) Research is not expected for my academic track  2) Research is expected for my academic track, but I have too many other responsibilities to focus on this aspect of my work  3) Research is expected for my academic track and my primary research focus is not in medical education.  4) Research is expected for my academic track and my primary research focus is in medical education, but I have other research opportunities, offered outside of COMSEP, that meet my needs | Strongly agree  Agree  Neither agree nor disagree  Disagree  Strongly disagree | 90 |
| 3) Exploring barriers to educational research  (explores contextual factors that may affect *personal agency*) | Think about the following 3 research offerings of COMSEP (Grants Program, Pediatrics feature, and the Annual Survey).  I have never submitted a proposal or pre-proposal to any of these 3 offerings: Grants Program, Pediatrics feature, and the Annual Survey. | YES/NO | 90 |
| 4a) Exploring barriers to educational research  (explores contextual factors that may affect *personal agency*) | SKIP LOGIC USED (if YES to Question 3, then)  Please help us understand why you have never submitted a proposal or pre-proposal by answering the following questions  1) Research is not of interest or importance to me  2) I don’t have the skills to do research  3) I don’t know how to get mentorship to take my research ideas to the next step  4) I do not enjoy writing grant proposals or manuscripts.  5) I have too many other responsibilities to make time for research | Strongly agree  Agree  Neither agree nor disagree  Disagree  Strongly disagree | 50 |
| 4b) Exploring satisfaction with and facilitators/barriers to educational research  (explores contextual factors that may affect *personal agency*) | SKIP LOGIC USED (if NO to Question 3, then)  Please help us understand your experience with the submission process and outcome.  1) I was satisfied with the submission process  2) I was satisfied with the review process  3) I was satisfied with the feedback I received | Strongly agree  Agree  Neither agree nor disagree  Disagree  Strongly disagree | 40 |
| 5) Organizational facilitators for research  (explores contextual factors that may affect *personal agency*) | We would like to understand if COMSEP could do something more or differently that would facilitate your participation in educational research.  Please rate the importance of the following actions COMSEP can consider taking to help you engage more effectively in educational research:  1) Offer more research or manuscript writing skill building opportunities  2) Offer personalized consultation on research ideas, submissions and pre-submissions  3) Make available consultants with research expertise to help with study design.  4) Increase funding opportunities for research  5) Improve how the current offerings are structured/implemented | Extremely important  Important  Unimportant  Extremely unimportant | 90 |
